# Role of marsupial tammar wallaby milk in lung maturation of pouch young

**DOI:** 10.1186/s12861-015-0063-z

**Published:** 2015-03-21

**Authors:** Vengamanaidu Modepalli, Lyn A Hinds, Julie A Sharp, Christophe Lefevre, Kevin R Nicholas

**Affiliations:** School of medicine, Deakin University, Pigdons Road, Geelong, Vic Australia; CSIRO Ecosystem Sciences, GPO Box 1700, Canberra, Act 2601 Australia

**Keywords:** Preterm birth, Lung maturation, Marsupials, Evolution, Lactation, Milk

## Abstract

**Background:**

Marsupials such as the tammar wallaby (*M.Eugenii*) have a short gestation (29.3 days) and at birth the altricial young resembles a fetus, and the major development occurs postnatally while the young remains in the mother’s pouch. The essential functional factors for the maturation of the neonate are provided by the milk which changes in composition progressively throughout lactation (300 days). Morphologically the lungs of tammar pouch young are immature at birth and the majority of their development occurs during the first 100 days of lactation.

**Results:**

In this study mouse embryonic lungs (E-12) were cultured in media with tammar skim milk collected at key time points of lactation to identify factors involved in regulating postnatal lung maturation. Remarkably the embryonic lungs showed increased branching morphogenesis and this effect was restricted to milk collected at specific time points between approximately day 40 to 100 lactation. Further analysis to assess lung development showed a significant increase in the expression of marker genes Sp-C, Sp-B, Wnt-7b, BMP4 and Id2 in lung cultures incubated with milk collected at day 60. Similarly, day 60 milk specifically stimulated proliferation and elongation of lung mesenchymal cells that invaded matrigel. In addition, this milk stimulated proliferation of lung epithelium cells on matrigel, and the cells formed 3-dimensional acini with an extended lumen.

**Conclusions:**

This study has clearly demonstrated that tammar wallaby milk collected at specific times in early lactation contains bioactives that may have a significant role in lung maturation of pouch young.

**Electronic supplementary material:**

The online version of this article (doi:10.1186/s12861-015-0063-z) contains supplementary material, which is available to authorized users.

## Background

Fetal lung development is a programmed cascade and any interruption in this process may cause abnormalities in neonates after birth [1]. The process of lung development in all mammals is similar [2] and is only different in the timing of development during perinatal and postnatal periods [3]. In eutherians, the placenta performs gaseous exchange between the fetus and the mother [4,5] and during gestation the lung matures and is capable of performing gaseous exchange after birth [6]. The main cause of respiratory complication in new born infants is due to pulmonary immaturity, known as respiration distress syndrome (RDS) and the main cause of RDS in preterm infants is lack of surfactant proteins in the lung [7-9].

During evolution of mammals, marsupials and eutherians have adopted different reproductive strategies which have given rise to differences in lung maturation at birth. Marsupials are placental mammals but they have a primitive form of placenta and give birth to immature young after a short gestation. The major growth and development of marsupial young occurs postnatally during early lactation [10]. The majority of eutherians, including the human, have a well-developed placenta and the lungs of neonates are at the alveolar stage of development [11]. In contrast, the lung in the marsupial newborn is immature and is still progressing from canalicular to early saccular stage of development [12]. The lung morphology of newborn marsupials, such as the short-tailed opossum (*Monodelphis domestica*) and tammar wallaby (*Macropus eugenii*), are comprised of large terminal air sacs with a small surface area for respiration [13]. Due to limited development of the neonatal lung the marsupial performs respiration through the skin to meet the demand for oxygen, and as the lung develops the total gaseous exchange is gradually shifted to the lung [14,15]. In all mammals, the extent of pulmonary development depends on the period of intrauterine development as the factors responsible for fetal development are provided through the maternal supply [16]. In marsupials the immature neonates rely on these maturation factors being provided through milk [17]. In eutherians at the end of gestation, the density of septal formation is high as the lung is progressing from saccular stages to the alveolar stage of development at birth. This septal division is carried out after birth to increase surface area for respiration by dividing large air sacs into alveoli. However alveolization requires necessary signalling factors for cell proliferation and function to produce surfactants and these are regulated by hormones in the newborn [18]. In contrast, the marsupial newborn lacks established functional hormonal systems [19,20]. In order to survive and develop they most likely need support from their mother through maternal milk [17].

Milk is composed of various bioactive components supporting early development of the neonate [21]. Eutherians, such as the human, undergo a long gestation followed by a relatively short lactation and the composition of milk does not change significantly [22,23]. In contrast, marsupials have a short gestation and a long lactation and milk composition changes significantly throughout lactation to provide factors for growth and development of the immature neonate [23,24]. The tammar wallaby is one of most studied marsupials and its lactation is divided into three phases (phase 2A, phase 2B and phase 3) based on the composition of the milk and growth and development of the young. Tammar young are born after 26 days of gestation and weigh approximately 440 milligrams [10]. During the early phase of lactation, the young remain permanently attached to the teat and the mother secretes dilute milk with a low concentration of protein and lipids but a high concentration of carbohydrates [25,26]. During the first 100 days the development of the neonate is similar to a late stage eutherian fetus and therefore the signalling factors involved in the development of the eutherian fetus are most likely delivered in the milk [27]. The pouch young are born with immature organs and during early lactation the organs necessary for their survival such as respiratory system [28], lymphoid tissues [29], nervous system including brain and spinal-card [30,31] are rapidly developed. Fostering experiments performed with tammars to understand regulatory effects of milk composition on rate of pouch young development, have demonstrated that by transferring the early pouch young to a late lactating tammar can accelerate the growth and physical development of pouch young [32,33]. Subsequent studies showed that cross fostering the young also accelerated maturation of specific organs such as the stomach [34]. Hence, the tammar provides an exceptional model for correlating milk composition with defined developmental changes in the immature lungs of the newborn as the respiratory process progresses from gas exchange across the skin to functional lungs during lactation. In this study we demonstrate the effect of bioactives in tammar wallaby milk collected in early lactation to accelerate growth and development of cultured mouse embryonic lungs.

## Results

### Postnatal development of lungs from tammar wallaby

**Figure 1:**
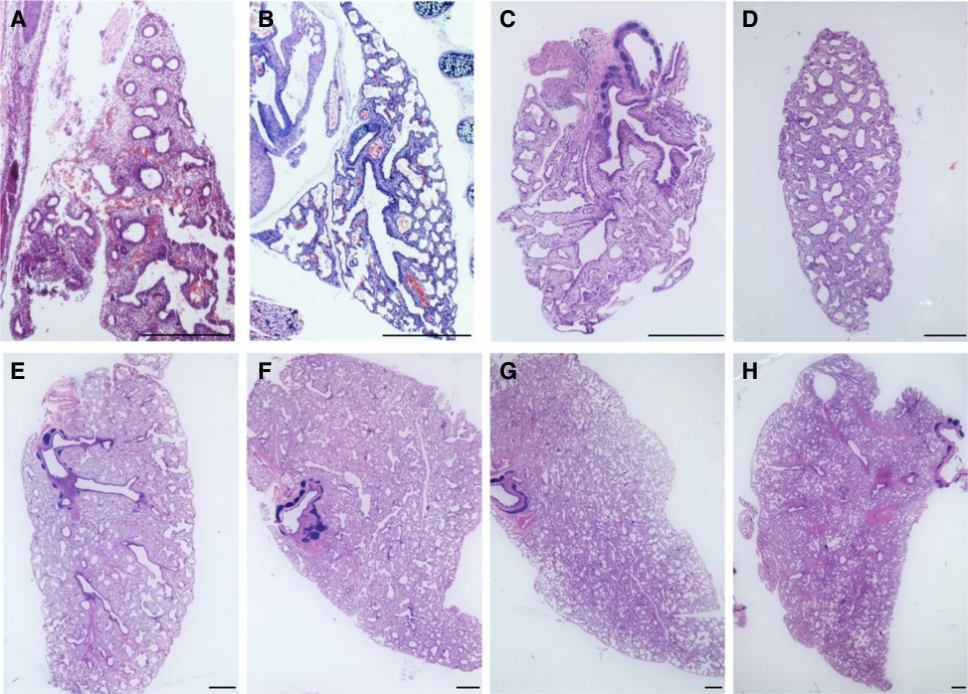
**Morphological development of tammar wallaby lung. (A-H)** Histological examination of H and E stained tammar pouch young lungs collected at **(A)** late gestation, ~ day 24; **(B)** postnatal day 1; **(C)** day 3; **(D)** day 20; **(E)** day 40: **(F)** day 60; **(G)** day 80 and **(H)** day 120 **(H)**. **(A)** Fetal lungs at the canalicular stage of development. **(B)** The lungs of newborn consist of large terminal air sacs surrounded by a thick wall. **(C,D)** During early postnatal life, lung development was slow, with large air sacs still present at day 20 **(D)**. **(E-H)** The alveoli-like structure appears by day 40 **(E)**, however at day 40 they appear quite primitive and are transiting from sac-like stature to alveoli. Defined alveoli start appearing from day 60 **(F)** and progressively increased in number thereafter **(F-H)**. Scale bar 1 mm.

Lungs of the near-term fetal tammar (~day 24) had primitive airways and canalicular like structures throughout (Figure 1A). Lungs of newborn tammar pouch young (PY) (Figure 1B) were immature with few terminal branches and large terminal air sacs with a thick wall, and the majority of tissue was comprised of mesenchyme. The lung at day 3 postpartum (Figure 1C) consisted of respiratory bronchioles terminated with large sac like structures. Around 20 days of age (Figure 1D) the large air sacs had undergone subdivision by formation of septa and the number of air sacs had increased. At this stage the majority of the lung was still comprised of sac like structures separated by connective tissue. The alveoli-like structure appears by day 40 (Figure 1E), however at day 40 they appear quite primitive and are transiting from sac-like stature to alveoli. Defined alveoli start appearing from day 60 (Figure 1F). By day 120, the lung had undergone further subdivision of air sacs and septa formation, alveoli-like structures had continued to form (Figure 1G). By day 120, the lung progressed to a mature morphology with enhanced alveolization (Figure 1H).

### Tammar wallaby day 60 milk stimulates development of cultured mouse embryonic lung

**Figure 2:**
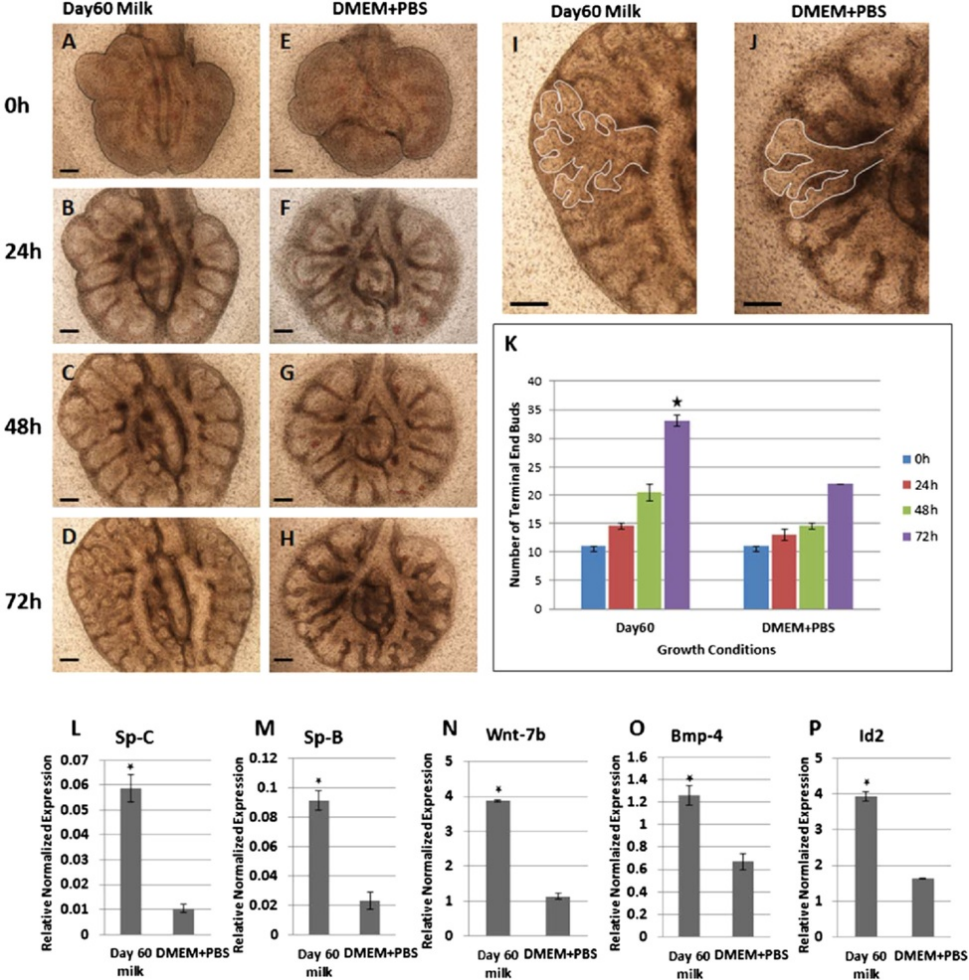
**Effect of tammar wallaby milk (day 60) on branching morphogenesis in cultured mouse embryonic lung.** Mouse E 12 lungs treated with tammar milk day 60 **(A-D)** or under control conditions **(E-H)** at (**A** and **E**) 0 hr (**B** and **F**) 24 hrs, (**C** and **G**) 48 hrs and (**D** and **H**) 72 hrs. **(I,J)** Increased branching morphogenesis shown by outlining terminal end buds. **(K)** Quantitation of branching morphogenesis at 0, 24, 48 and 72 hrs shows number of terminal end buds of tammar day 60 milk treated is greater than control treated lungs. Expression of critical developmental marker genes **(L)** Sp-B & (M) Sp-C (Type-II pneumocytes marker genes), **(N)** wnt-7b, **(O)** BMP4 and **(P)** Id-2 in mouse embryonic lung cultured for 72 h with or without day 60 tammar milk. Scale bar 250 μm. P values (<0.05) are shown with an asterisk.

To determine the potential effect of marsupial milk in lung development, embryonic lungs from mice were cultured in serum free media plus 10% tammar whey collected at day 60 of lactation (Figure 2A-D). In control cultures the embryonic lungs were incubated in serum free media with 10% PBS (Figure 2E-H). The embryonic lungs exposed to day 60 tammar milk whey showed extensive branching morphogenesis and increased volume over 3 days of culture (Figure 2I). In contrast, the control embryonic lungs showed a relative delay in branching morphogenesis and the lung was smaller after 3 days of the culture (Figure 2J). The number of terminal end buds significantly increased over 3 days in the lungs treated with day 60 milk (Figure 2K). The level of expression of several developmental marker genes was assessed by using q-PCR analysis. The developmental marker genes SP-C (Figure 2L) and SP-B (Figure 2M) (Type-II pneumocytes), Wnt-7b (Figure 2N) and BMP-4 (Figure 2O) (branching morphogenesis), and Id-2 (Figure 2P) each showed a significant increase in levels of expression relative to control lungs (*P* < 0.05). Further, embryonic lungs were cultured for 4 days in media with 10, 5 & 2.5% of day 60 milk protein. To determine the effect of concentration of tammar milk on lung growth. No significant difference in number of terminal end buds was observed in tissue cultured in media with milk protein concentrations of 10%, 5% and 2.5%. Lungs cultured in media with each concentration of milk protein showed similar epithelial branching and vascular development which was significantly advanced when compared to lungs cultured in control media (see Additional file 1: Figure S1).

### Tammar wallaby milk proteins from early lactation differentially regulate embryonic mouse lung development

**Figure 3:**
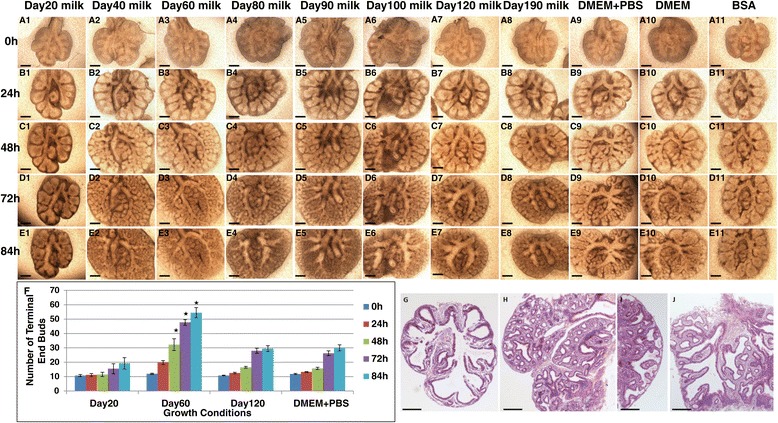
**Tammar milk regulates the growth of mouse embryonic lungs in culture.** Embryonic lungs were cultured for 86 h in presence of wallaby milk collected at day 20 **(A1-E1)**, day 40 **(A2-E2)**, day 60 **(A3-E3)**, day 80**(A4-E4)**, day 90 **(A5-E5)**, day 100 **(A6-E6)**, day 120 **(A7-E7)** day 190 **(A8-E8)**, control embryonic lung cultured in media with PBS **(A9-E9)**, media **(A10-E10)** and in media with BSA **(A11-E11)**. **(A1-E1)** Embryonic lung treated with day 20 milk showed no change in number of terminal end buds. Embryonic lungs with day 40 **(A2-E2)**, day 60 **(A3-E3)**, day 80 **(A4-E4)**, day 90 **(A5-E5)** and day 100 **(A6-E6)** milk showed branching after 48 h. **(A7-E7)** Embryonic lung cultured with day 120 **(A7-E7)** and day 190 **(A8-E8)** milk showed branching after 72 h in culture, but the total volume of lung remained unchanged. Control embryonic lung cultured in media with PBS **(A9-E9)** and media only **(A10-E10)** showed delayed branching and no change in lung volume. **(A11-E11)** Embryonic lung cultured in media with BSA showed no effect on branching. **(F)** Analysis of branching morphogenesis by counting the number of terminal end buds of tammar milk treated day 20, day 60, day 120 and control embryonic lung. **(G)** Histological analysis of mouse embryonic lung section stained with H&E after culture with day 20 tammar milk. A small number of terminal end buds were present; **(H)** day 60 milk – a large number of terminal end buds and well developed branching morphogenesis is evident. **(I)** day120 milk - fewer terminal end buds and increased mesenchyma, and **(J)** Control (PBS) - extended branching is present, but the numbers of terminal end buds were less when compared to embryonic lungs treated with day 60 milk **(B)**. Scale bar 250 μm. p values (<0.05) are shown with an asterisk.

To examine whether the signalling factors in day 60 milk responsible for lung development were secreted in other phases of lactation, mouse embryonic lung was incubated with milk proteins collected from day 20, day 40, day 60, day 80, day 90, day 100 day 120 and day 190 of lactation (Figure 3). Embryonic lungs (n ≥4) were cultured for 4 days in media with either 10% milk protein, 10% PBS or 10% bovine serum albumin (BSA). Digital images of lung explants were examined every 24 hours and the number of terminal end buds determined (Figure 3U). In control experiments where explants were cultured in media with either PBS (Figure 3 A9-E9) or media alone (Figure 3 A10-E10), the majority of the explant was populated with long tubules with poor branching morphogenesis. A significant reduction in end bud formation was evident and explants showed low branching morphogenesis until the end of 4 days of culture. In control experiments where embryonic lungs were cultured in media with BSA (Figure 3 A11-E11) and media that included day 20 tammar milk (Figure 3 A1-E1) the explants showed significant reductions in branching morphogenesis and the volume of lung remained similar to that observed at time zero. Embryonic lung cultured in media with phase 2B tammar milk collected on day 120 (Figure 3 A7-E7) and day 190 (Figure 3 A8-E8) of lactation had commenced branching morphogenesis by day 3 in culture and there was no increase in total volume of lung, similar to that observed in embryonic lungs cultured in day 20 milk. These explants showed no significant increase in number of terminal end buds in comparison with control lung. In the explants treated with day 40 (A2-E2), day 60 (A3-E3), day 80(A4-E4), day 90 (A5-E5) and day 100 (A6-E6) milk, the stimulatory effect on branching was more prominent with lung tissue showing branching morphogenesis after 48 h of culture and an accompanying increase in the number of terminal end buds when compared to the control explants. By the end of culture at day 4 the number of terminal end buds showed a gradual increase in lung cultured with day 40–100 milk and further, the terminal end buds increased by 86 h in embryonic lungs treated with day 60 milk when compared with control embryonic lung. Mouse embryonic lung explants treated with day 20 milk had rudimentary branches and did not change during 4 days of culture (Figure 3G). In contrast, explants treated with day 60 milk showed significant increases in branching of the main bronchus to form putative alveolar regions in the majority of the lung (Figure 3H), and peripheral epithelial tubules showed a complex branching pattern in comparison with control explants. Explants treated with day 120 milk (Figure 3I) had small epithelial tubules with terminal end buds at peripheral regions. In lungs cultured in control media the majority of each explant was populated with long tubules with poor branching morphogenesis and the absence of epithelial sacs (Figure 3J).

### Developmental marker gene expression in cultured mouse embryonic lungs

**Figure 4:**
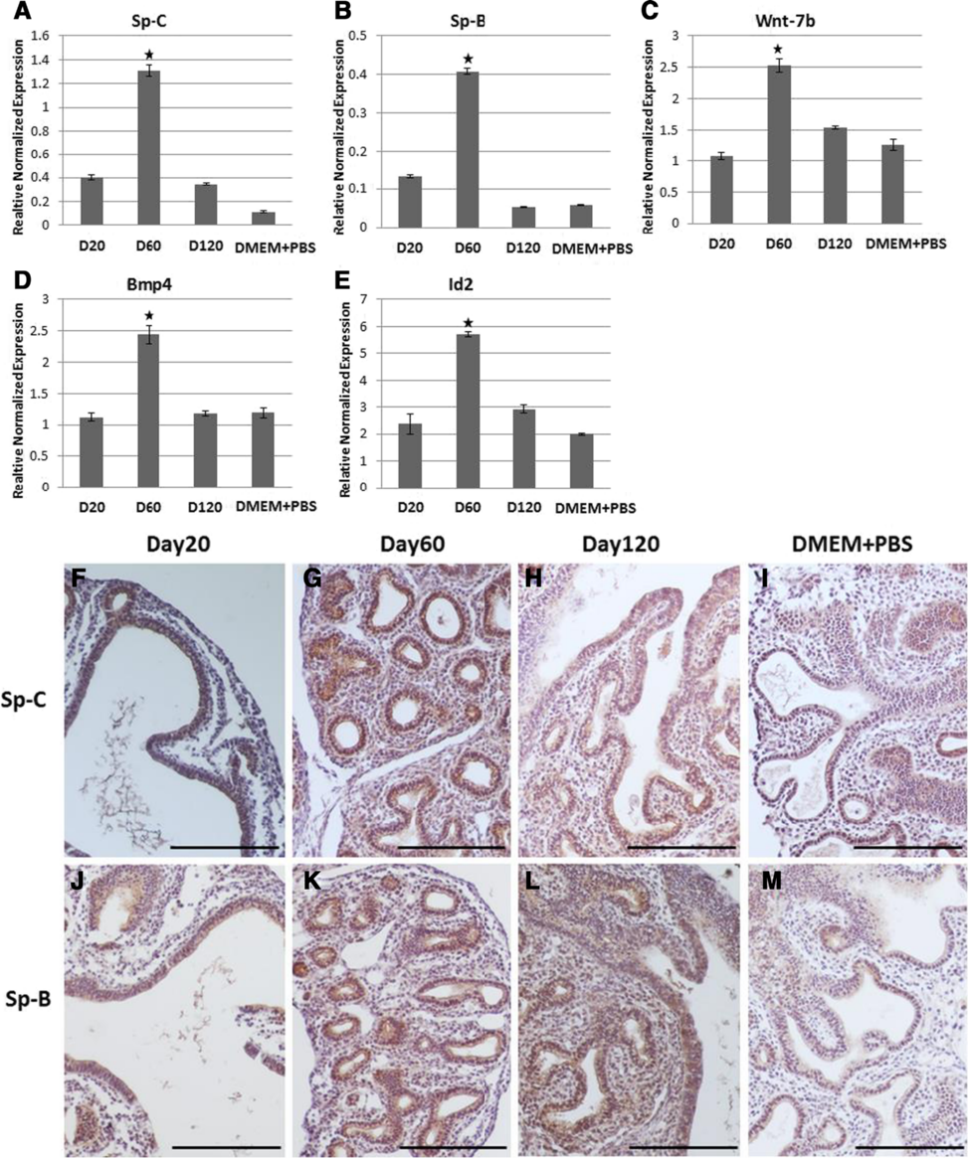
**Expression of lung developmental marker genes in mouse embryonic lung cultured with tammar milk.** RNA was isolated from embryonic lungs cultured for 84 h to observe the expression of **(A)** *Sp-C* & **(B)** *Sp-B* (type-II cell marker gene), **(C)** *wnt-7b*, **(D)** *BMP4* and **(E)** *Id-2* marker genes. **(A-E)** qRT-PCR of Sp-C, Sp-B, wnt-7b, BMP-4 and Id-2 mRNAs were increased in embryonic lung cultured with tammar milk with statistically significant P values (<0.05) shown with an asterisk. Immunohistochemical analysis of **(F-I)** Sp-C and **(J-M)** Sp-B in cultured embryonic lung. Lung sections from embryonic lung treated with tammar milk day 20 **(F,J)**, day 60 **(G,K)**, day 120 **(H,L)** and control **(I,M)** were immunostained with type-II cell marker surfactant protein C and DAB was used for visualization. **(G,K)** The Sp-C protein is observed in high concentration in embryonic lungs treated with day 60 milk. **(I,M)** In control lungs Sp-C protein is detected at low levels. Scale bar 100 μm.

Expression of the marker genes *SP-C, SP-B, Wnt-7b*, *BMP-4* and *Id-2* were examined in mouse embryonic lung explants treated with media that included either 10% day 20, day 60, day 120 milk or a control that included 10% PBS in media. The embryonic lungs treated with day 60 milk showed a significant increase in the level of expression of all marker genes in comparison with control embryonic lung (Figure 4). Expression of surfactant Sp-C and Sp-B protein was examined in embryonic lungs treated with day 20, day 60, day 120 milk and control lungs using immunohistochemistry (Figure 4). Sp-C was highly localised to acinar tubules and terminal end buds of lungs but Sp-B protein was only detected in acinar tubules. Surfactant proteins Sp-C (Figure 4G) and Sp-B (Figure 4K) were detected at high levels in embryonic lungs treated with day 60 milk. However, both surfactant proteins were also detected in embryonic lungs treated with milk protein day 20 & day 120 (Figure 4).

### Effect of tammar milk on mouse embryonic lung epithelium and mesenchymal cell proliferation

**Figure 5:**
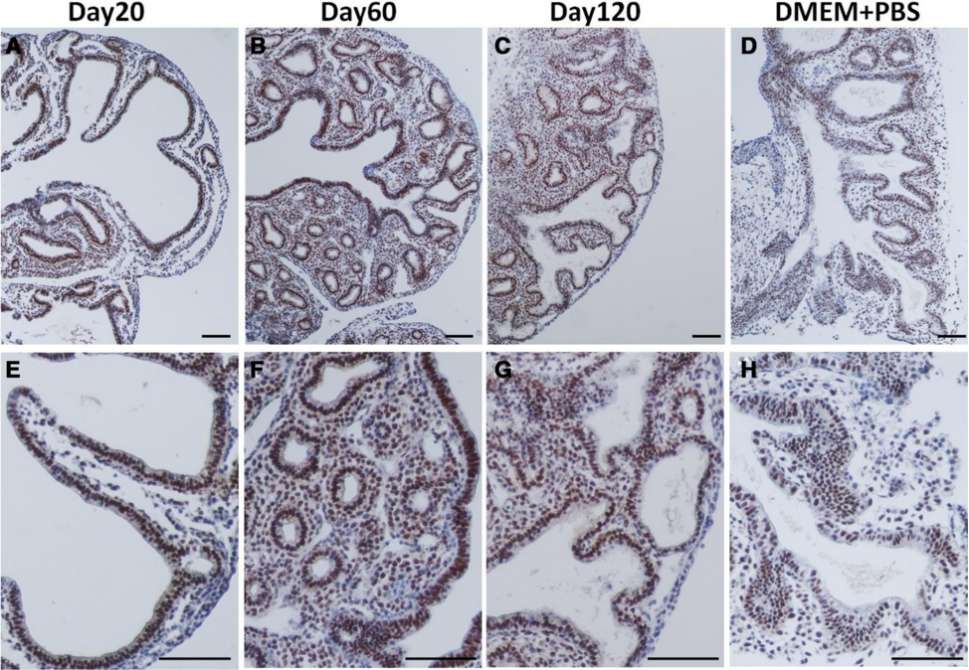
**PCNA Cell proliferation immunostaining of cultured embryonic lungs. (A-C)** Embryonic lungs treated with tammar milk and **(D)** control. Cell proliferation in both mesenchyme and epithelium was detected by immunostaining with PCNA antibody and counterstained with haematoxylin. **(E-H)** Higher magnification views of A-D. **(A,E)** Embryonic lung treated with day 20 milk showed low cell proliferation in both epithelium and mesenchyma. **(B,F)** Embryonic lung treated with day 60 milk showed cell proliferation in both epithelium and mesenchyma around the terminal end buds. **(C,G)** Embryonic lung treated with day 120 milk showed cell proliferation in both mesenchyma and epithelium. The distribution of mesenchyma was increased in explants treated with day 60 **(B,F)** and 120 **(C,G)** milk when compared to explants treated with day 20 **(A,E)** milk. **(D,H)** Lung cultured in control media showed a low level of cell proliferation. Scale bar 100 μm.

Proliferating cell nuclear antigen (PCNA) staining was used to examine the rate of cell proliferation in mouse embryonic lung mesenchyme and epithelium (Figure 5). The PCNA immunostaining of embryonic lungs cultured in media with day 20 milk showed strong immunostaining in terminal end bud epithelium and low level staining in the mesenchyme (Figure 5A). In contrast, embryonic lungs treated in media with day 60 (Figure 5B) and day 120 (Figure 5C) milk showed an increase in the ratio of stained mesenchymal cells to epithelial cells.

### Effect of tammar milk on isolated mouse embryonic lung epithelium in matrix

**Figure 6:**
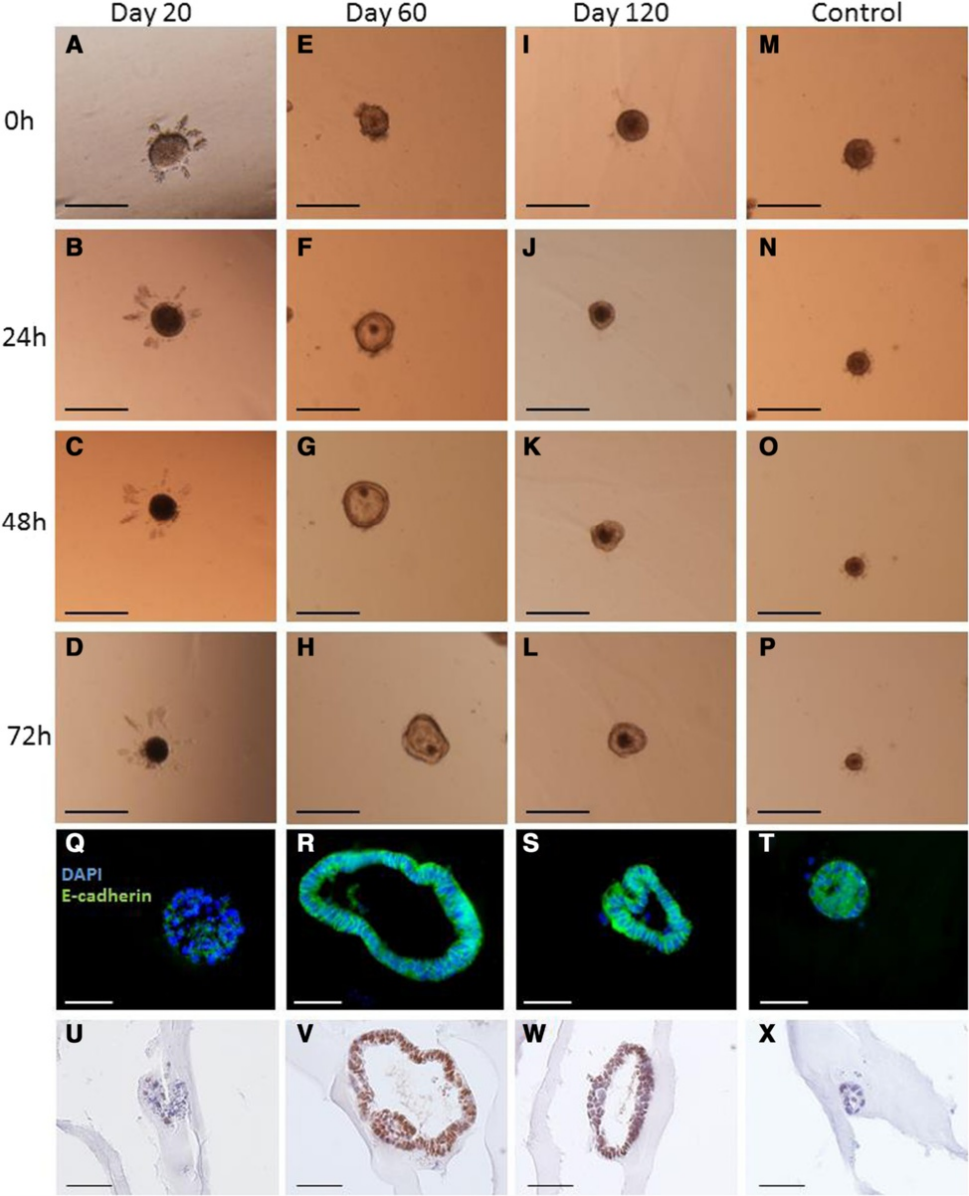
**Bright field images of embryonic lung epithelial 3D explants cultured for 3 days in matrix. (A-D)** Explants cultured in the presence of day 20 milk, **(E-H)** day 60 milk, **(I-L)** day120 milk and **(M-P)** in the absence of milk. **(Q-T)** The effect of tammar milk on the morphology of embryonic lung epithelial explants. Epithelial membranes were visualised by staining with E-cadherin (green) and cell nuclei with Dapi (blue). **(Q)** Explants treated with day 20 milk showed a disorganized cell mass and no lumen. **(R)** Explants treated with day 60 milk show a large lumen surrounded by columnar epithelial cells. **(S)** Explants treated with day 120 milk show a small lumen lined with columnar epithelial cells. **(T)** Explants cultured in the absence of milk showed no defined lumen and a disorganized cell mass. **(U-X)** Cell proliferation of epithelial explants was observed by immunostaining with PCNA antibody (brown) and counterstained with haematoxylin (blue). **(U)** Explants treated with day 20 milk showed minimal cell proliferation in comparison to explants treated with day 60 milk **(V)**, and almost all cells stained with PNAS. **(W)** Embryonic lung treated with day 120 milk showed a reduced number of cells stained with PCNA compared to explants cultured in day 60 milk. **(X)** In explants in control media there was no evidence of PCNA staining in the cells. **(Q-X)** Scale bar 100 μm, **(A-P)** Scale bar 200 μm.

**Figure 7:**
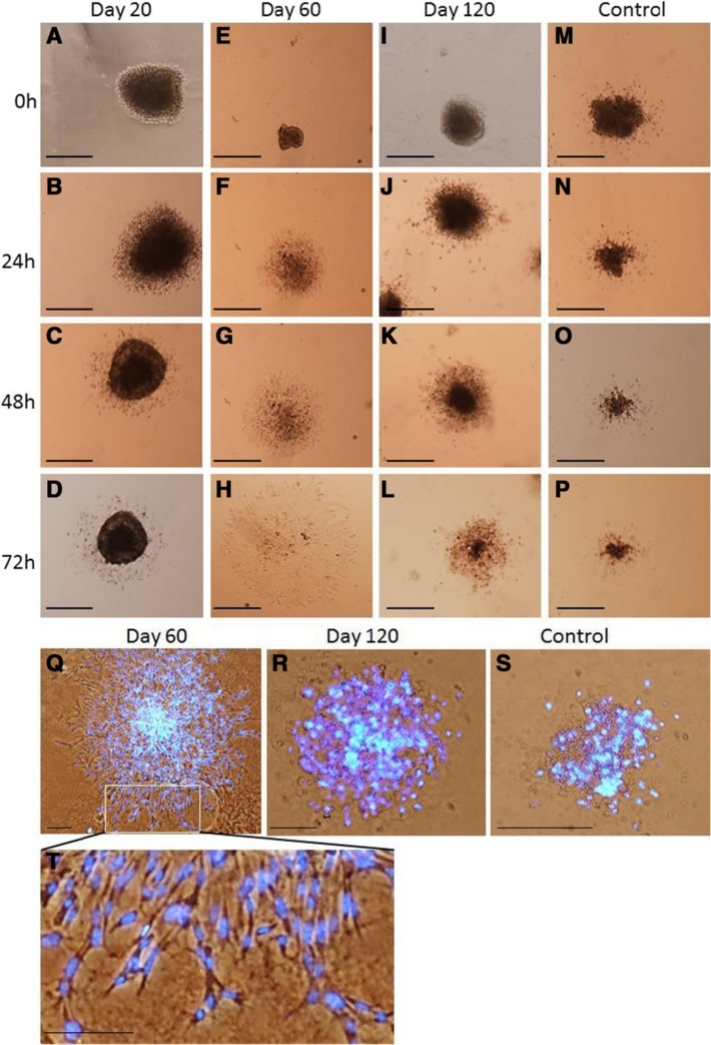
**Bright field images of 3D embryonic lung mesenchymal explants cultured for 3 days in matrix. (A-D)** Explants cultured in media with day 20 milk showed cell clumping with no significant changes over time. **(E-H)** Explants cultured in the presence of day 60 milk showed an increase in cell number and cell differentiation. **(I-L)** Explants cultured in the presence of day 120 milk showed cell shrinkage and apparent necrosis and a large reduction in cell population. **(M-P)** Explants cultured as controls in media but in the absence of milk. **(Q-T)** Immunofluorescence of embryonic lung mesenchymal explants in matrix culture. **(Q)** Explants cultured in the presence of day 60 milk and stained with DAPI (blue) showed increased cell number and cell differentiation. **(T)** Cells shown in **(Q)** at higher magnification. **(R)** Explants cultured with day 120 milk. **(S)** Explants cultured as controls in the absence of milk. The cell debris and large reduction in cell population was similar in explants cultured with day 120 milk and control explants. Scale bar 200 μm.

To determine whether the effect of tammar milk targeted either lung epithelium or mesenchyma, embryonic epithelium and mesenchymal cells were cultured for 3 days in Matrigel with media that included milk collected from tammars at day 20, day 60 and day 120 of lactation (Figure 6 and 7). The size of epithelial explants treated with day 20 milk decreased over the 3 day period (Figure 6D) and immunofluorescence staining showed epithelium was aggregated with disorganized cell debris (Figure 6Q). PCNA staining showed epithelium treated with day 20 milk had insignificant proliferation (Figure 6U). Epithelial explants cultured with day 60 milk appeared larger in size with a large lumen (Figure 6H). Immunofluorescence staining showed no cell mass in the centre of explants and the lumen was surrounded by simple columnar epithelial cells (Figure 6R) and a large number of cells were PCNA positive (Figure 6V). Explants in day 120 milk had a small cell mass in the lumen (Figure 4L), increased lumen formation (Figure 6S) and increased cell proliferation consistent with PCNA staining (Figure 6W). The epithelial explant treated with day 120 milk was comparatively smaller than observed following culture with day 60 milk (Figure 6R,S). In the absence of tammar milk, control explants showed no significant growth in size (Figure 6M-P). Necrotic cells were observed in the centre of explants surrounded by cells but epithelial cells showed minor cell proliferation (Figure 6T).

### Effect of tammar milk on isolated mouse embryonic lung mesenchyma in matrix

To investigate whether there was a direct effect of tammar milk on lung mesenchyma, the isolated mesenchyma from E12 embryonic mouse lung was cultured for 3 days as a 3D explant in matrigel and treated with tammar milk (day 20, day 60 and day 120). Control mesenchymal explants, cultured in media with no milk proteins, initially aggregated and did not develop further exhibiting cell shrinkage indicative of necrotic cells after 3 days of culture (Figure 7 M-P). The mesenchyma treated with day 20 milk showed no growth and became condensed (Figure 7A-D). The mesenchyma treated with day 60 milk showed morphological cell differentiation and proliferation and elongated mesenchymal cells invaded the matrigel (Figure 7E-H). Whole mount staining of these explants demonstrated the cells were flat, elongated and spindle shaped similar to either airway smooth muscle cells or myofibroblast cells derived from primary mesenchyma (Figure 7Q&T). Mesenchymal explants treated with day 120 milk (Figure 7I-L) were similar to control cultures (Figure 7 M-P) with cells failing to survive and condensing with apparent necrosis. Whole mount staining of these explants indicated likely necrosis and a large reduction of cell population (Figure 7R&S).

## Discussion

### Tammar lactation is programmed to potentially regulate lung development in pouch young by stimulating branching morphogenesis and growth

The tammar is born after a short gestation (~28 days) and lungs are immature at birth with the majority of development occurring during early postnatal life [35]. The in depth analysis of tammar lung development described here showed fetal lungs were still at the canalicular stage of development during the final stages of gestation (~day 24 gestation) similar to eutherian fetal rat at ~ E14 gestation [31,36]. The lungs at birth were at a transitional stage from a canalicular to saccular structure with the formation of primitive air sacs and most of the lung was comprised of mesenchyme tissue. In contrast, in the majority of eutherian species the lung develops prior to birth, while respiration is supported by the maternal placenta [16]. Unlike eutherians, the marsupial newborn has immature organs, including the lung [35] and most of their early development relies on factors supplied through milk [17]. Interestingly, the delay in gut maturation is considered advantageous in neonatal mammals like marsupials, as their gastro-intestinal system is immature during early lactation and it has the capacity to absorb maternal proteins like immunoglobulins in milk through the gut [37]. Indeed, absorption of macromolecules persists throughout most of pouch young life [38,39]. The current study examined whether marsupial tammar wallaby milk includes bioactives with the potential to stimulate branching morphogenesis in lung. Mouse embryonic lungs were cultured with tammar milk protein and results showed a significant effect on branching morphogenesis and growth of lung explants treated with day 60 tammar milk.

Analysis of the pattern of gene expression in the tammar wallaby mammary gland has shown that the majority of genes are differentially expressed during lactation [17]. Moreover there is a gradual change in the number of milk proteins secreted across lactation with the potential to influence development and immune protection of pouch young [40-43]. Previous studies have shown that transfer of tammar pouch young to a mother at a more advanced stage of lactation can specifically accelerate the rate of stomach development in pouch young [44] and the current study is consistent with the hypothesis that tammar lactation may also be programmed to regulate postnatal lung development during early lactation after the first 40 days postpartum. The potential inhibitory effect of milk at day 20 on *in vitro* lung development correlates with the time when the early saccular phase of development is evident and the lung displays a slow rate of septal formation. This may result from milk that includes a negative regulator or potentially that the milk is lacking necessary signals for branching morphogenesis and increased lung volume. However, this observation correlates with the time when a significant proportion of respiration occurs through the skin of the pouch young [35]. In tammar pouch young the majority of lung development was observed around day 40 as the lung progressed from saccular stage to alveolar stage. During this process the septal crest density increased from ~ day 40 – ~ day 100 and was observed as the most active state of alveolarization in relation to growth of lung. At this time lungs progress from saccular to alveolar (day 40) stage although a significant increase in alveolar number was observed until the end of phase 2A (day 100 lactation). This profound increase in branching morphogenesis indicates the milk at day 40 – day 100 may have the necessary bioactivity to regulate lung development in pouch young and correlates with the timing of increased bioactivity of milk in the *in vitro* lung model. Further, morphological changes observed in tammar pouch young lung after phase 2A lactation at day 120 clearly indicate a slow rate of development of septal density which is consistent with the effects induced by milk from day 120 and day 190 of lactation when there was very limited increase in terminal branches and growth in cultured mouse lungs.

RNA analysis on mouse embryonic lung explants treated with milk from day 20, day 60 and day 120 of lactation were analysed for expression of a group of marker genes to assess lung development in culture; surfactant proteins (SP-C & SP-B) and branching morphogenesis proteins (BMP-4 & Wnt7b). Surfactant proteins SP-C and SP-B are the two major surfactant proteins expressed in the early developmental stage of the lung [45]. SP-C is involved in postnatal respiration [46] and SP-B is involved in immune protection [47]. Increased expression of these surfactant proteins in mouse lung explants treated with day 60 milk indicate that factors in milk were able to stimulate proliferation of Type-II epithelial cells and increase synthesis of surfactant proteins. Expression of surfactant protein is essential for successful respiration in newborns and decreased expression of surfactant proteins may cause respiratory distress syndrome in preterm infants [7].

The early stage of lung development involves increased branching morphogenesis from the main bronchus to increase surface area for gaseous exchange [9,48]. Expression of BMP-4 and Wnt7b are involved in branching morphogenesis during embryonic lung development [49,50]. Terminal end buds from the primary branches progress to terminal air sacs [51], a process associated with increased epithelial cell proliferation [52]. Tammar milk was shown to include bioactives that increase terminal end buds and signal epithelial cell proliferation and branching morphogenesis when cultured with lungs isolated from mouse embryos. A significant increase in terminal end buds was observed in lungs treated tammar milk collected specifically at day 40 - day 100 of lactation and the increase in the level of expression of the BMP-4 and Wnt7b genes is consistent with tammar milk proteins activating branching morphogenesis. These data suggest that milk secreted during early lactation may have the potential to signal branching morphogenesis of altricial young in the pouch. Escalated branching morphogenesis supports the increase of total volume of the lung providing the required area for lung alveolarization in later stages of development, which in turn provides required surface areas for respiration after birth. In addition, during this process in cultured embryonic lungs there are progenitor cells located in terminal end buds that can differentiate into epithelial cells in the alveolar and bronchial regions. Expression of the Id2 gene is a marker for these progenitor cells [53] and expression of the Id2 gene was increased in embryonic lungs treated with tammar milk day 60, but not in lung explants treated with milk from early (day 20), late lactation and under control conditions. Further, cell proliferation was higher in embryonic lung treated with day 60 milk and the increased rate of cell proliferation observed in mesenchyme in embryonic lungs treated with day 120 milk indicates that these changes most likely results from increased mesenchymal signalling factors.

### Tammar milk protein regulates the growth of mouse lung epithelium and mesenchyma

The current study showed a difference in the ratio of epithelium and mesenchyme in embryonic lung when treated with tammar milk protein collected from different time points. Subsequent experiments showed that tammar milk stimulated epithelium and mesenchyma separately to regulate cell differentiation, proliferation and polarity of the cells. Epithelial explants specifically treated with day 60 milk preparation developed a large lumen surrounded with a single layer of columnar epithelial cells. As mesenchymal-epithelial interactions are essential for epithelial branching morphogenesis [54,55]. The addition of day 60 milk stimulated the cell behaviour of a primitive mesenchyma, with an increase in cell proliferation and elongation of mesenchymal cells invading the surrounding matrigel. The role of day 60 milk in mesenchymal cell proliferation and differentiation is still unclear, however, observation of cells during culture showed they were flattened, elongated and spindle shaped, representing either airway smooth muscle cells or myofibroblast cells derived from primary mesenchyma [56]. A decrease in mesenchymal tissue can lead to a delay or prevention of epithelial branching during lung development [55,57]. In contrast, the treatment of lung with day 20 milk showed a reduced effect on epithelium and mesenchymal cell populations which is consistent with a similar effect observed when the whole embryonic lung was cultured in day 20 milk protein. Therefore, factors in milk responsible for postnatal lung maturation were expressed from day 40 of lactation until the end of the early lactation period (phase-2A) and this temporal effect was lost in later phases of lactation (phase 2B & phase 3). The timing of this stimulatory activity of milk on mouse embryo lungs is consistent with increased lung development in tammar neonates and reduced level of lung development after phase 2A lactation.

## Conclusion

Taken collectively the results presented in this study indicate that tammar milk can temporally regulate cell proliferation and differentiation of both epithelium and mesenchyma cell populations from lung. This concept of the timed presentation of milk bioactives to the young for lung development is consistent with the timed appearance of milk bioactives that regulate gut development in the young and protection of the young from infection [33,43,44]. This unique model may offer new opportunities for the identification of signalling molecules that are presented to the marsupial young at a time that correlates with prenatal presentation of signalling factors by the placenta and amniotic fluid for the development of a range of tissues during eutherian foetal development.

## Methods

### Ethical approval

A colony of tammar wallabies (*M. eugenii*) was maintained at Deakin University, Geelong, Victoria, Australia and at CSIRO Ecosystem Sciences, Canberra, Australia. All animal experimentation was approved by The Deakin University and CSIRO Animal Ethics committees.

### Histological analysis of tammar wallaby lung during postnatal development

Lung tissues were collected from tammar young’s at late gestation, ~ day 24 and postpartum day 1, 3, 20, 40, 60, 80 and 120 (n = 3-5 young’s per state). The age of pouch young was either determined from known birth dates or estimated by measuring head length [58]. The lung was removed, rinsed thoroughly with sterile PBS and fixed overnight in 4% formaldehyde prior to paraffin embedding. Tissue sections (5–6 μm) were prepared and stained with haematoxylin and eosin (H&E) for examination of morphological development.

### Milk collection and processing

Tammars were anaesthetised with Isoflurane and 0.2 IU of Oxytocin-S® (Intervet, Boxmeer, The Netherlands) was administered intramuscularly prior to milk collection. Milk was collected from each animal by applying gentle pressure to the mammary glands and milk was stored at −80°C until further analysis. Milk was obtained from mothers at 20, 40, 60, 80, 90, 100, 120 & 190 days of lactation. Milk samples were thawed on ice and centrifuged at 5000xg to isolate the fat and cells. Skim milk was centrifuged at 17,000Xg for 10 min to remove the majority of casein. The whey was sterilised using a 0.22 μm filter (Corning Costar Spin-X Centrifuge Tube Filters) and samples were aliquotted and stored at −80°C until further analysis.

### Mouse embryonic lung isolation and culture

A total of 32 C57BL/6 wild-type mice were killed at E12.5 pregnancy and the lungs were excised from 108 embryos. After removal from the uterus, the embryos were washed in Dulbecco’s Phosphate Buffered Saline (DPBS) and transferred to a Petri dish with Hanks’ Balanced Salt solution (HBSS) placed on ice. Embryonic lungs dissected from each embryo were transferred to Transwells with serum free Dulbecco’s Modified Eagle’s Medium/F12 (DMEM/F12) media that included 1% Penicillin and streptomycin [59], and cultured in media with 10% whey prepared from milk collected at different stages of wallaby lactation. Control lungs were cultured in media with 10% PBS only. Lung explants were incubated at 37°C, 5% CO_2_ for 3–4 days and the media and milk protein was replaced every 24 h. Due to less quantity availability of day 20 milk the treatment was carried out on 4 embryonic lungs. The remaining treatments were performed approximately 10–14 embryonic lungs and the experiments were performed at least 3 times.

### Culture of embryonic lung epithelium and mesenchyma

Lungs were isolated from the E-12 mouse embryos. The lungs were treated with dispase (1.5 U/ml DPBS) at 37°C for 30 min to separate the epithelium and mesenchymal tissue. The distal mesenchyma was separated from the epithelial bud by using a pair of tungsten needles and a dissection microscope [59]. Geltrex® Matrix (Life Technologies^TM^) was prepared by 1:1 dilution with culture media and transferred to a culture dish with separated epithelium and mesenchyma. Matrix together with cells was allowed to polymerize at 37°C for 1 hr. After polymerization 1 ml of DMEM/F12 media that included 1% Penicillin, 1% streptomycin and 1% Fetal Bovine Serum (FBS) was added and the cells incubated at 37°C in 5% CO2. The cells were cultured in media with 10% wallaby whey (as above) and the control lungs were cultured in media with 10% PBS only. The explants were cultured for 3 days and the images were collected at 24 hr intervals.

### Morphological analysis of embryonic lung

At the completion of culture lung explants were washed twice with PBS, fixed in 4% formaldehyde in PBS and embeded in paraffin. Lung sections (5-6 μm) were stained with H&E to examine lung morphology. Branching morphogenesis of lung explants after different treatments was quantified by counting the number of terminal end buds around the circumference of each explant. For each treatment a minimum of four embryonic lungs were analysed. Results were analysed using a one Tailed, Type-2 T-test to estimate the statistical significance and the error bars indicate sample ± SEM.

### Total RNA isolation and quantitative RT-PCR

Total RNA was extracted from lung tissue using a PureLink® RNA Mini Kit (Life Technologies) and following the manufacturer’s instructions, and subsequently quantified by spectrophotometry (Nano drop ND-1000, Biolab, VIC, Australia). First-strand cDNA was synthesised using Superscript III^TM^ Reverse Transcriptase (Invitrogen), following the manufacturer’s instructions. Quantitative RT-PCR (qPCR) was performed using the SsoFast EvaGreen Supermix (Bio-Rad) and CFX96TM Real-Time PCR Detection System (Bio-Rad). The PCR reaction (20 μL) contained 1× master mix, 0.25 μM of forward and reverse primers (see Additional file 2: Table S1) and diluted cDNA template. All samples were assayed in triplicate. Amplification curves were generated with an initial denaturing step for 30 minutes at 94°C, followed by 40 cycles at 94°C for 30 seconds, 60°C for 30 seconds and 72°C for 30 seconds. The GAPDH gene was used as an internal control.

### Immunohistochemistry

The cultured embryonic lungs were immediately fixed in 4% formaldehyde, dehydrated and embedded in paraffin. Sections (5-6 μm) were prepared and deparaffinised. The sections were treated with 3% H_2_O_2_ and the antigen retrieval was achieved in 10 mM citrate buffer (PH 6.0). Immunohistochemistry of surfactant proteins SP-C and SP-B was performed using primary antibody Goat anti-SftpC (1:200, Santa Cruz, (C-19): sc-7705) and Rabbit anti-Pro SftpB (1:600, Abcam, ab15011). A Streptavidin-HRP conjugated Rabbit anti-Goat IgG anti-HRP (1:200, Bethyl, A50-100p) and Goat anti-Rabbit HRP (1:100, Abcam, ab97051) secondary antibody and the DAB substrate kit (DAB Substrate Kit, Cell Signaling, #8059) was used for detection. The nuclear counter stain was performed using haematoxylin.

### Cell proliferation

Paraffin embedded embryonic lungs and epithelial explants were sectioned (5–6 μm) and the mesenchyme and epithelial cell proliferation was measured using a PCNA kit (PCNA staining kit, Invitrogen, 93–1143) following the manufacturer’s instructions. The number of PCNA positively stained cells in both mesenchyme and epithelium was counted in random parts of a section by using Image J software.

### Analysis of 3D epithelium and mesenchyma culture by immunofluorescence staining

Lung epithelial explants were collected after 3 days of culture and prepared for paraffin sectioning. The slides were processed for staining as described above. The epithelial sections were incubated overnight with E-cadherin (1:400, Cell Signaling Technology, 24E10) primary antibody. The sections were washed with PBS and incubated with secondary antibody conjugated with Alexa Fluor® 488 (1:400, Life Technologies). Finally all samples were mounted with Dapi mounting media (Fluoroshield Mounting Medium with DAPI, Abcam, ab104139) prior to photography with fluorescence microscopy. For whole-mount staining the mesenchyma in matrix were fixed in 4% formaldehyde for 1 hr and stained for nuclei using Hoechst (1:1000).

## Additional files

Additional file 1: Figure S1. Effect of the concentration of tammar milk in media on embryonic lung growth stimulation. The embryonic lungs were cultured with either 10%, 5% and 2.5% tammar milk protein collected at day 60 of lactation or control media (Figure 1A). Branching morphogenesis was quantitated by counting the number of terminal ends of embryonic lung (Figure 1B). There was no significant difference observed among embryonic lungs treated with 10% and 2.5% of tammar milk. Additional file 2: Table S1. Primer sequences used for mRNA quantification by RT-PCR.
