# Supplementary figures and images for: Role of marsupial tammar wallaby milk in lung maturation of pouch young
Source: BMC Dev Biol. 2015 Mar 21;15:16. doi: 10.1186/s12861-015-0063-z (PMC4377010; doi:10.1186/s12861-015-0063-z)

## Slide 1
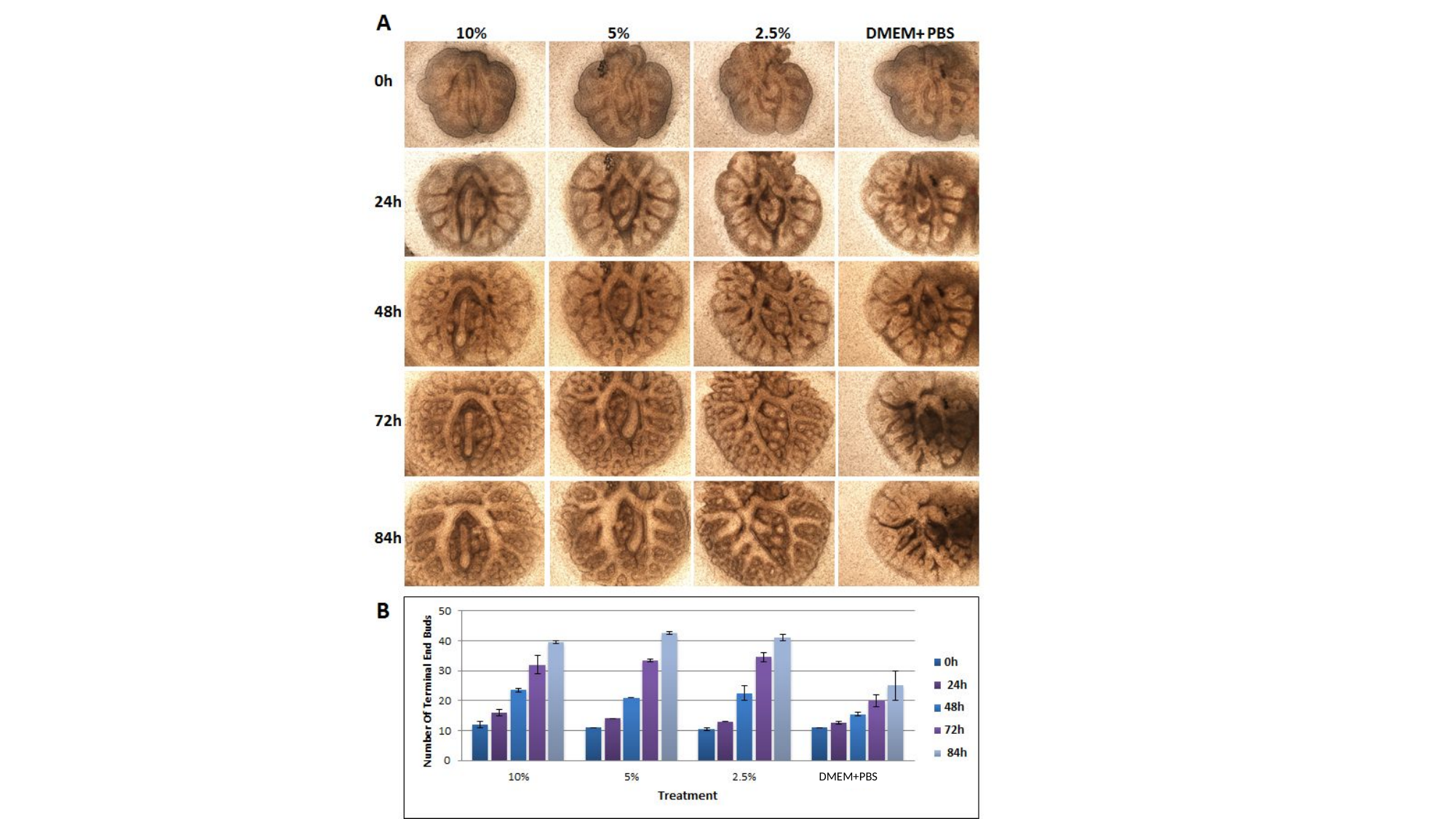

DMEM+PBS

Supplement: Additional file 1: Figure S1. — Effect of the concentration of tammar milk in media on embryonic lung growth stimulation. The embryonic lungs were cultured with either 10%, 5% and 2.5% tammar milk protein collected at day 60 of lactation or control media (Figure 1A). Branching morphogenesis was quantitated by counting the number of terminal ends of embryonic lung (Figure 1B). There was no significant difference observed among embryonic lungs treated with 10% and 2.5% of tammar milk. [file 12861_2015_63_MOESM1_ESM.pptx]
