# Supplementary material for: Role of marsupial tammar wallaby milk in lung maturation of pouch young
Source: BMC Dev Biol. 2015 Mar 21;15:16. doi: 10.1186/s12861-015-0063-z (PMC4377010; doi:10.1186/s12861-015-0063-z)
Supplement: Additional file 2: Table S1. — Primer sequences used for mRNA quantification by RT-PCR. [file 12861_2015_63_MOESM2_ESM.pdf]

Supplement table 1. Primer sequences used for mRNA quantification by RT-PCR

Gene	Forward Primer sequence	Reverse Primer sequence
**SPC**	5′-GGACATGAGTAGCAAAGAGG-3′	5′-GTAGAGTGGTAGCTCTCCAC-3′
**SPB**	5′-CTGCTGGCTTTGCAGAACTC-3′	5′-GGTTTGGAAGCACTGCAGAG-3′
**WNT7B**	5′-ACGGCATCGACTTTTCTCGT-3′	5′-AGGTCCGTCTCCATAGGCT-3′
**BMP4**	5′-TCCATCACGAAGAACATC-3′	5′-TAGTCGTGTGATGAGGTG-3′
**ID2**	5′-CTCCAAGCTCAAGGAACTGG-3′	5′-ATTCAGATGCCTGCAAGGAC-3′
**GAPDH**	5′-TGAACGGGAAGTCACTGG-3′	5′-TCCACCACCCTGTTGCTGTA-3′
